# Mild maternal sleep-disordered breathing during pregnancy and offspring growth and adiposity in the first 3 years of life

**DOI:** 10.1038/s41598-020-70911-4

**Published:** 2020-08-19

**Authors:** Avivit Brener, Yael Lebenthal, Sigal Levy, Galit Levi Dunietz, Orna Sever, Riva Tauman

**Affiliations:** 1 Pediatric Endocrinology and Diabetes Unit, Dana-Dwek Children’s Hospital, Tel Aviv Sourasky Medical Center, 6423906 Tel Aviv, Israel; 2 Sackler Faculty of Medicine, Tel Aviv University, 6997801 Tel Aviv, Israel; 3 Statistical Education Unit, The Academic College of Tel Aviv Yaffo, 6818211 Tel Aviv, Israel; 4 Sleep Disorders Center, Department of Neurology, University of Michigan, Ann Arbor, MI USA; 5 Sleep Disorders Center, Tel Aviv Sourasky Medical Center, 6 Weizman Street, 6423906 Tel Aviv, Israel

**Keywords:** Endocrinology, Medical research

## Abstract

Sleep-disordered breathing (SDB) during pregnancy has been linked to adverse fetal outcomes. Since the intrauterine milieu plays a critical role in childhood growth, we explored the interactions between maternal SDB and offspring growth and adiposity patterns during infancy. Fifty-eight healthy women with uncomplicated pregnancies underwent an objective sleep study and laboratory evaluation during the third trimester, their offspring underwent a 3-year growth surveillance. The 14 (24.1%) women with SDB had a higher body mass index (BMI) (*P* = 0.003), elevated C-reactive protein levels (*P* = 0.003), and decreased HDL-cholesterol levels (*P* = 0.009) than the women without SDB. A general linear model evaluated the interactions between maternal SDB and offspring growth and adiposity measurements after controlling for gestational age and maternal and paternal BMIs. The offspring of mothers with SDB had a significantly smaller head circumference at birth (*P* = 0.004), with a distinctive pattern of catchup growth by the end of the first year of life (*P* = 0.018). Their growth pattern was distinguished by compromised birth weight-to-length, rapid catch-up growth, and an increase in both weight-to-length and triceps thickness by the age of three (*P* < 0.001 and *P* = 0.001, respectively). Our findings suggest that maternal SDB during pregnancy affects head circumference growth and adiposity acquisition from birth through infancy.

## Introduction

Growth and development during infancy and childhood are affected by both genetics and environment. The intrauterine milieu dictates not only fetal biometric parameters but also growth and development patterns during childhood. A growing body of evidence suggests that maternal morbidities during pregnancy have unfavorable implications on childhood anthropometry. Maternal insulin resistance during pregnancy was related to a higher offspring body mass index (BMI) at 3 years of age^1^, maternal hypertension during pregnancy was associated with both offspring obesity at 4–7 years^2^ and higher blood pressure^3^, and altered maternal inflammatory status was linked to early childhood overweight^4^. It stands to reason that other gestational conditions could also have an impact on the newborn's growth and adiposity.

Sleep-disordered breathing (SDB) is a condition of abnormal respiration during sleep that ranges from primary (habitual) snoring to obstructive sleep apnea syndrome. It is characterized by episodic complete or partial obstruction of the airway during sleep, disruption of normal ventilation, intermittent hypoxemia, and sleep fragmentation. Physiologic changes occurring during pregnancy, particularly during the third trimester, place women at risk for developing SDB^5,6^. Similar to the general population, overweight and obesity also increase the risk of SDB in pregnant women^7^. An association has been reported between SDB during pregnancy and gestational diabetes mellitus, hypertension, and preeclampsia^8–16^. In addition, altered fetal growth, prematurity, cesarean section, and low Apgar scores have been reported in pregnant women with SDB, although the findings were inconsistent^16–25^. In the general population, SDB is associated with metabolic consequences, such as insulin resistance, dyslipidemia, and type 2 diabetes^26^. Thus, it is possible that gestational SDB changes the metabolic milieu of the fetus as well, leading to alterations in its growth patterns and adiposity.

We had recently shown that maternal SDB—even in its mild form—is associated with accelerated fetal growth that is expressed in several dimensions of growth, such as weight, length, and adiposity^25^. In the present report, we aimed to analyze the offspring's growth pattern and adiposity acquisition as monitored throughout the first 3 years of life.

## Methods

### Study population

**Figure 1:**
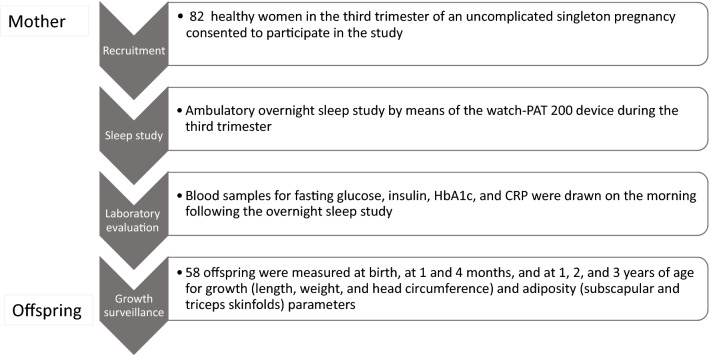
Flowchart of the study protocol.

Healthy pregnant women in the third trimester of a spontaneous uncomplicated singleton pregnancy, who were followed at the low-risk obstetric surveillance clinic at the Noy Women Health Care Center or the outpatient clinics at the Lis Maternity Hospital were recruited between April 2013 and May 2016. Participants were followed from the third trimester of pregnancy until delivery and their offspring were followed from birth until 3 years of age. Exclusion criteria were documented diagnoses of pregnancy, fetal, obstetric or pediatric complications (including gestational hypertension, chronic maternal medication administration, gestational diabetes mellitus, fetal intrauterine growth restriction, congenital anomalies, infections, birth asphyxia and significant pediatric morbidity or chronic medication administration which may affect growth). The study protocol is presented in Fig. 1. Women diagnosed as having SDB comprised the study group and those without SDB comprised the control group. Informed consent was obtained from all participants. The study was carried out in accordance with GCP guidelines and was approved by the Institutional Review Board of the Tel Aviv Medical Center (IRB No. 0510-12-TLV).

### Data collection

All participants completed a questionnaire at enrollment that included maternal age, maternal weight and height before pregnancy and at the third trimester, paternal weight and height, past and present maternal medical history, previous pregnancy complications, and smoking before and during pregnancy. In addition, maternal medical files were reviewed for possible maternal and/or fetal complications^27^. BMI was calculated as weight in kilograms divided by height in meters squared. Following delivery, obstetric and labor records as well as newborn records were reviewed for gestational age, infant birth weight, Apgar scores at 1 and 5 min after birth, and documentation of any infant physical abnormalities.

### Sleep study

All participating women were requested to undergo an ambulatory overnight sleep study during the third trimester (between 33 to 36 weeks of gestation) that was conducted by means of the watch-PAT 200 device (Itamar Medical; Israel) which has been validated in pregnancy and shown to correlate well with polysomnography^28^. An apnea hypopnea index (AHI), mean oxygen saturation (SpO_2_) and nadir SpO_2_ levels were retrieved as described in detail elsewhere^29^. Women with an AHI equal to or greater than 5 per hour of sleep were considered to have SDB^30^. Mild SDB was defined as being within a range of 5 to 15/h.

### Laboratory evaluation

Blood was drawn from all participants on the morning following the overnight sleep study for fasting glucose, insulin, hemoglobin A1c (HbA1c), lipid panel (total cholesterol, triglycerides, LDL-cholesterol, HDL-cholesterol) and C-reactive protein (CRP). Homeostatic Model Assessment of Insulin Resistance (HOMA-IR) was calculated according to the formula: fasting glucose level (mg/dl) × fasting insulin level (μU/ml)/405^31^. The healthy range for HOMA-IR in non-pregnant women was defined as being between 0.5 to 1.4, early insulin resistance as a value above 1.9, and significant insulin resistance as a value above 2.9. There are no published data on HOMA-IR cutoff levels in pregnant women. We interpreted the results according to accepted HOMA-IR thresholds in non-pregnant women, since previous studies reported that HOMA-IR values in women with normal glucose tolerance did not change significantly with advancement of pregnancy^32,33^.

### Anthropometric measurements of offspring from birth to 3 years of age

Growth (length, weight and head circumference) and adiposity (subscapular and triceps skinfolds) parameters were measured at birth, at 1 and 4 months, and at 1, 2, and 3 years of age. The anthropometric measurements were conducted by the same trained medical personnel throughout the entire study period. Weight was measured by an electronic infant scale (Model 20 Tabletop Infant Scale, Olympic Medical, Seattle, WA). Recumbent crown-heel length was measured in the supine position on a length board (O’Leary Premie Length Board, Ellard Instrumentation Ltd., Monroe, WA). Weight-to-length ratio was calculated as an indicator for weight status in children younger than 3 years of age^34^. Fronto-occipital head circumference was measured using a standard 1-cm wide measuring tape. Standard deviation scores (SDS) were calculated for length, weight, weight-to-length ratio and head circumference using the growth chart percentiles of the Centers for Disease Control and Prevention in order to compare anthropometric parameters across age groups by sex^35^. Skinfold thickness measurements were performed using Holtain calipers (Holtain, UK). Triceps skinfold was measured over the posterior belly of the triceps muscle of the extended right arm halfway between the acromion and the olecranon, on a line passing upwards from the olecranon in the axis of the limb. Subscapular skinfold was measured immediately below the angle of the right scapula in the natural cleavage line of the skin, with the arm held by the side of the body. Skinfold measurements were made by lifting the full thickness of skin with thumb and index finger. Each skinfold measurement was recorded twice and the mean was used for analysis^36,37^. At each scheduled growth assessment visit the mothers reported whether their offspring is being/was breastfed (yes/no).

### Statistical analysis

Descriptive statistics are presented as mean (SD) or count (%) as applicable. Independent samples t-test and the Chi-square test were used to compare the distributions of maternal, newborn, and early childhood characteristics of offspring among women with mild SDB (AHI within a range of 5 to 15/h) compared with controls. We examined the interaction between maternal AHI and offspring growth and adiposity measures over time by constructing a general linear model with time as a within-subject factor and with group as a between-subject factor. The model was controlled for gestational week at delivery, maternal BMI, and paternal BMI. A non-parametric Spearman's correlation test was used to assess the correlation between duration of breastfeeding and growth parameters. Data were analyzed using the IBM SPSS software (IBM SPSS Statistics for Windows, Version 25, Armonk, NY: IBM Corp.). A *P* value of ≤ 0.05 was considered significant.

## Results

Eighty-two women and their offspring were recruited to the study; of those, 58 offspring completed 3 years of growth surveillance (Fig. 1). Comparative analysis of maternal characteristics of the participants who completed the study (n = 58) and those who dropped out (n = 24) showed no significant differences between the two groups in any parameter. None of the participants had maternal- or pregnancy-related complications, such as hypertension, gestational diabetes, intrauterine infection, or fetal growth restriction. The mothers' mean age at recruitment was 33 ± 4.2 (range 25 to 43.5 years), and 28 (48.3%) were primiparous. The mean maternal BMI was 23.3 ± 4.0 before pregnancy and 28.0 ± 3.9 at delivery. The mean paternal BMI was 24.5 ± 2.8 (range 16.7 to 33.9).

Fourteen (24.1%) of the women were diagnosed as having SDB with a mean AHI score of 7.6 (range 5.3 to 14.7, all within the 5 to 15/h range indicative of the mild form of SDB). The characteristics of mothers stratified by the presence of SDB is presented in Table 1. Women with SDB had a higher BMI before pregnancy compared to women without SDB (26.0 ± 4.1 vs. 22.5 ± 3.6, respectively, *P* = 0.003), while the delta BMI between the end-pregnancy and the pre-pregnancy BMI was similar between the two groups (4.1 ± 1.7 vs. 3.6 ± 5.7, *P* = 0.749). There was no significant difference in maternal age or proportion of primiparous women between the groups. Maternal laboratory evaluations revealed elevated CRP levels in mothers with SDB (6.39 ± 2.29 mg/L vs. 4.28 ± 2.15 mg/L for those without SDB, *P* = 0.003). Mothers with SDB had lower HDL-cholesterol levels (67 ± 14 mg/dl vs. 82 ± 19 mg/dl, respectively, *P* = 0.009), without a significant difference in total cholesterol, LDL-cholesterol, and triglycerides levels. Glycemic parameters (HbA1c and HOMA-IR) did not differ between groups.

**Table 1:** Maternal characteristics, sleep study and laboratory results of the 58 study participants stratified according to maternal sleep-disordered breathing (SDB).

Variable	SDB	No SDB	*P*
Number (%)	14 (24.1)	44 (75.9)	
Maternal age, years	34.2 (4.1)	32.6 (3.9)	0.192
Primiparous, n (%)	4 (28.6)	24 (54.5)	0.094
Maternal pre-pregnancy BMI	26.0 (4.1)	22.5 (3.6)	**0.003**
Maternal end-pregnancy BMI	30.1 (3.9)	27.2 (3.5)	**0.011**
Past smoking, n (%)	3 (21.4)	10 (22.7)	0.920
Pregnancy smoking, n (%)	2 (14.3)	1 (2.3)	0.081
**Sleep study**			
AHI	7.6 (2.3)	1.4 (1.4)	** < 0.001**
SpO_2_ nadir	90.7 (1.5)	92.6 (2.7)	**0.015**
SpO_2_ mean	95.7 (0.9)	95.8 (1.0)	0.740
**Laboratory evaluation**			
HOMA-IR	2.6 (1.2)	2.7 (1.7)	0.839
HbA1c, %	5.3 (0.4)	5.1 (0.4)	0.109
CRP, mg/L	6.4 (2.3)	4.3 (2.2)	**0.003**
Total cholesterol, mg/dL	235 (32)	255 (45)	0.129
Triglycerides, mg/dL	259 (107)	226 (63)	0.160
LDL-cholesterol, mg/dL	123 (34)	129 (35)	0.576
HDL-cholesterol, mg/dL	67 (14)	82 (19)	**0.009**

Data are presented as mean (standard deviation) unless otherwise specified.

Bold indicates significant.

BMI, body mass index; AHI, apnea hypopnea index; SpO_2_, oxygen saturation; HOMA-IR, Homeostatic Model Assessment of Insulin Resistance; HbA1c, hemoglobin A1c; CRP, C-reactive protein; LDL-cholesterol, low-density lipoprotein-cholesterol; HDL-cholesterol, high-density lipoprotein-cholesterol.

**Figure 2:**
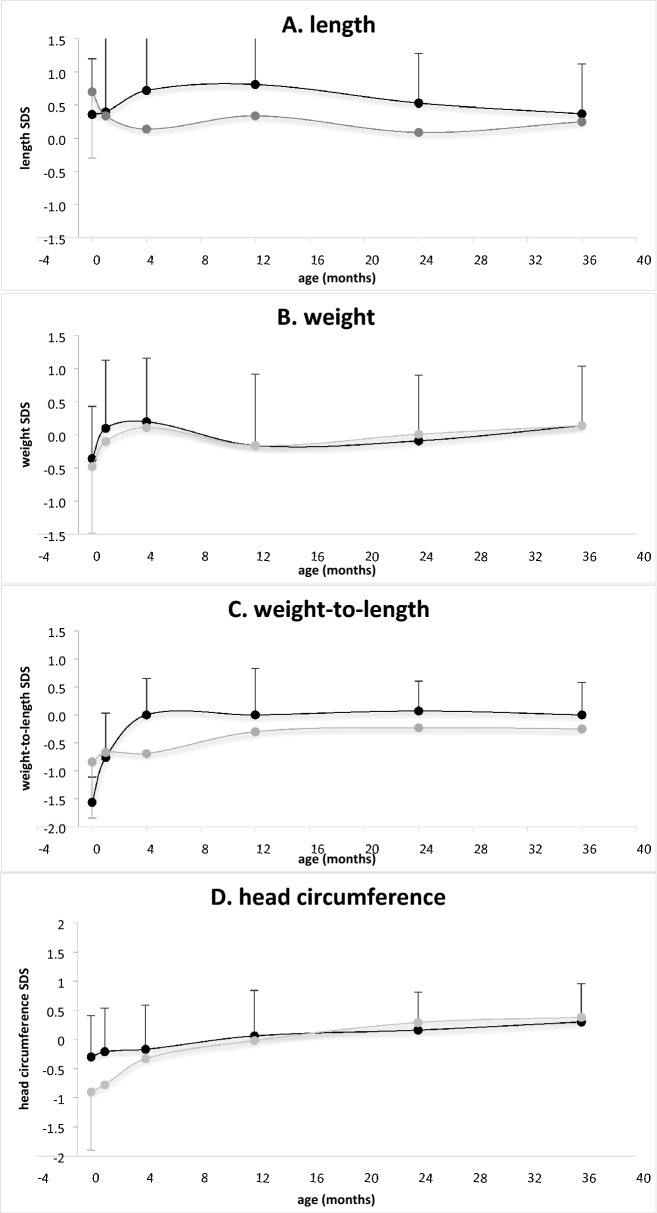

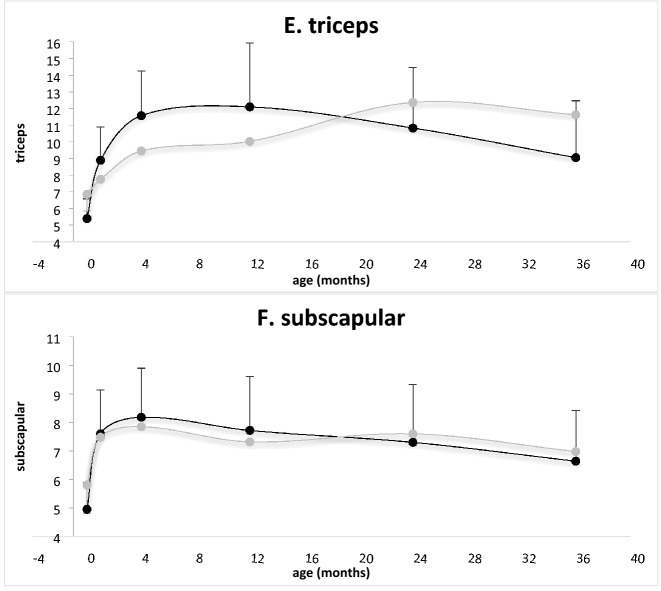
Longitudinal anthropometric and adiposity measures of offspring: (**A**) length, (**B**) weight, (**C**) weight-to-length ratio, (**D**) head circumference (data are presented as mean Z score), (**E**) triceps, and (**F**) suprascapular skinfolds (data are presented in millimeters). Light grey represents offspring of mothers with mild SDB, black represents offspring of mothers without SDB.

All newborns were born at term (mean gestational age 39.4 ± 1.5 weeks), 33 (56.9%) were males, the mean birth weight SDS was − 0.39 ± 0.75, the mean birth body length SDS was 0.43 ± 0.89, the mean birth weight-to-length ratio SDS was − 1.00 ± 1.31 and the mean birth head circumference SDS was − 0.39 ± 0.74. Characteristics of the offspring stratified by the presence of maternal SDB are presented in Table 2. Longitudinal growth parameters (length SDS, weight SDS, weight-to-length ratio SDS and head circumference SDS) and longitudinal adiposity measures (triceps and subscapular skinfolds) stratified by the presence of maternal SDB are presented in Fig. 2.

**Table 2:** Characteristics of the offspring at birth according to the presence of maternal sleep-disordered breathing (SDB).

Characteristic	SDB	No SDB	*P*
Male sex, n (%)	10 (71.4)	23 (52.3)	0.213
Gestational age weeks	39.2 (1.4)	39.4 (1.5)	0.661
Apgar 1 min, median (range)	9 (7 to 9)	9 (8 to 9)	
Apgar 5 min, median (range)	10 (8 to 10)	10 (8 to 10)	
Birth weight SDS	− 0.48 (0.64)	− 0.36 (0.79)	0.608
Birth length SDS	0.57 (0.76)	0.36 (0.84)	0.409
Birth weight-to-length ratio SDS	− 1.56 (0.45)	− 0.84 (1.17)	**0.029**
Head circumference SDS	− 0.95 (0.70)	− 0.30 (0.71)	**0.004**
Subscapular skinfold, mm	5.8 (1.3)	5.0 (1.0)	**0.019**
Triceps skinfold, mm	6.8 (1.8)	5.4 (1.2)	**0.002**

Data are presented as mean (standard deviation) unless otherwise specified. SDS, standard deviation score.

Bold indicates significant.

A general linear model revealed a significant interaction over time between maternal SDB and growth of head circumference [F(5, 270) = 2.8, *P* = 0.018], between maternal SDB and weight-to-length ratio [F(5, 255) = 3.4, *P* < 0.001], and between maternal SDB and adiposity acquisition as measured by triceps skinfold [F(5, 195) = 4.0, *P* = 0.002], after controlling for other covariates (gestational age, maternal BMI, and paternal BMI). There was no significant interaction between maternal SDB and other anthropometric parameters (length and weight) or subscapular skinfold thickness after controlling for the same covariates.

A non-parametric Spearman's correlation yielded no significant correlation between breastfeeding duration and growth parameters, therefore breastfeeding duration was not included in the statistical model. One-way analysis of variance revealed no significant differences in growth and adiposity measures between the breastfed and non-breastfed infants.

## Discussion

This is the first study to report that mild maternal SDB during pregnancy in otherwise healthy women with uncomplicated singleton pregnancies exerts a negative effect on their offspring’s head circumference growth in utero, with a distinctive growth pattern during the first 3 years of life. In addition, our findings suggest that offspring of mothers with mild SDB had a compromised weight-to-length ratio at birth, rapid catch-up growth, and an increased weight status with increased adiposity acquisition in the first 3 years of life.

We found that the women with mild SDB during pregnancy had higher BMI levels and were relatively overweight before pregnancy. Their weight gain during pregnancy was similar to that of women without SDB, contradictory to the recommendations of lower weight gain during pregnancy in overweight women^38^. This finding suggests that maternal weight gain during pregnancy in women who were relatively overweight before pregnancy may play a role in the development of SDB. The maternal lipid profile was characterized by significantly lower HDL-cholesterol levels in women with mild SDB, without any difference in other lipoproteins. Low HDL-cholesterol is a known atherogenic risk factor and a component of metabolic syndrome^39^, and lower HDL levels may be early evidence of risk for metabolic syndrome in mothers with SDB. The lack of elevated triglyceride and LDL-cholesterol levels highlights the milder nature of the metabolic disturbance in these women.

The mothers with SDB had elevated CRP levels. Elevation in proinflammatory cytokines secondary to intermittent hypoxia and oxidative stress was reported by Ryan et al.^40^. Poor sleep quality and continuity were also associated with elevated CRP levels in healthy young non-pregnant women^41^. Increased inflammatory markers, such as elevated interleukin 6 (IL-6), tumor necrosis factor-α, and CRP protein levels, were described in adverse pregnancy outcomes, and increased IL-6 levels were additionally associated with maternal snoring^42^. The inflammatory response could, therefore, be the missing link between our findings of maternal mild SDB during pregnancy and long-term effects on child growth and adiposity.

We found that mild maternal SDB was associated with the offspring's smaller head circumference at birth, and that those infants demonstrated a distinctive pattern of head circumference growth during the first 3 years of life. The relatively smaller head circumference, albeit within the normal range, in the first months of life with rapid catchup growth by the end of the first year of life may have long-term implications. A recent report documented that the growth of head circumference is a surrogate marker of brain development, and that atypical head circumference growth is associated with neurodevelopmental disorders^43^. Conditions affecting the intrauterine environment were linked to maternal hormonal changes; even mild and transient changes capable of affecting the expression of target genes were reportedly involved in brain growth and maturation^44,45^. Elevated maternal CRP levels during pregnancy were reported in association with variable medical conditions affecting the brain, such as autism^46^, but the actual impact of mild SDB associated with mild CRP elevation on cognitive function and behavior is unknown. In this study we did not perform neurological assessment, therefore clinical meaning of our finding remains to be determined. In a previous study we reported the detection of a low social developmental score at 1 year of age in 64% of the infants born to SDB mothers compared to 26% of infants born to non-SDB controls, suggesting that even mild maternal SDB may affect social development at 1 year^47^. Our results taken together with those of other reports highlight the importance of neurodevelopmental assessment in the offspring of mothers with SDB.

The newborns of mothers with mild SDB had compromised weight status at birth, as expressed by weight-to-length ratio, with rapid catchup growth during the first 4 months of life to a relatively increased weight status. At birth, weight status was decreased while adiposity measured by skinfolds thickness was increased. This discrepancy could be attributed to a distinctive fat distribution with increased peripheral fat and decreased abdominal fat. Offspring's longitudinal adiposity acquisition revealed increased adiposity at 3 years of age, as measured by triceps skinfold. Of note, this difference could not be explained by paternal or maternal BMI or by duration of breastfeeding. Surveillance of weight status and adiposity measures during childhood and adolescence is warranted. Multiple perinatal insults were identified as independent risk factors for numerous medical conditions, including obesity, in human adults^48^, however, the impact of subtle maternal medical conditions, such as mild SDB, has yet to be determined. Animal studies reported increased adiposity in adult mice exposed to intermittent hypoxia during prenatal life, however, this model does not mimic mild SDB in humans^49^.

In our cohort, women with mild SDB had lower SpO_2_ nadir measurements. Maternal hypoxia has been shown to induce epigenetic changes with long term affects on offspring development and health^50,51^. It is plausible that maternal oxygen desaturation, although very mild, contributed to the distinctive growth patterns of the offspring observed in our study. Notwithstanding such considerations, thresholds for maternal SpO_2_ affecting the fetus have yet to be determined^52^.

The major strength of our study is its prospective nature and the relatively large number of participants in the third trimester of pregnancy who completed objective maternal sleep studies, the results of laboratory evaluations, and surveillance of the offspring’s growth over time, including adiposity measurements for the first 3 years of life. Another strength is the performance of anthropometric and adiposity measurements by the same trained medical personnel along the entire study period. The major limitation of this study is the lack of serial neurocognitive assessments which could provide a more comprehensive evaluation of child development. The attrition rate (29%) is also a drawback: only 58 of 82 of the offspring completed 3 years of growth surveillance. Another limitation is the lack of data on mothers' ethnicity and socioeconomic position which may affect fetal and neonatal growth outcome^53^.

In conclusion, the intrauterine environment affects growth and adiposity acquisition, and the effect is first manifested in birth parameters that are reflected in fetal growth and echoes along early childhood. Our findings also suggest that maternal mild SDB during pregnancy affects the growth of head circumference and adiposity parameters during the first 3 years of life. The clinical implications of these findings on neurocognitive development and adiposity in childhood and adolescence during critical periods of growth have yet to be elucidated and warrant investigation. Mild SDB is an underdiagnosed medical condition that affects a substantial proportion of healthy women. Early diagnosis and appropriate intervention in mothers may prevent future adverse consequences to their offspring.

## References

[1] Finnbogadóttir SK (2017). Insulin resistance in pregnant women with and without polycystic ovary syndrome, and measures of body composition in offspring at birth and three years of age. Acta Obstet. Gynecol. Scand..

[2] Zheng JS (2017). Maternal blood pressure rise during pregnancy and offspring obesity risk at 4 to 7 years old: The Jiaxing Birth Cohort. J. Clin. Endocrinol. Metab..

[3] Falkner B (2020). Maternal and gestational influences on childhood blood pressure. Pediatr. Nephrol..

[4] Englich B (2017). Maternal cytokine status may prime the metabolic profile and increase risk of obesity in children. Int. J. Obes..

[5] Pien GW, Schwab RJ (2004). Sleep disorders during pregnancy. Sleep.

[6] Izci B (2003). The upper airway in pregnancy and pre-eclampsia. Am. J. Respir. Crit. Care Med..

[7] Leung PL, Hui DSC, Leung TN, Yuen PM, Lau TK (2005). Sleep disturbances in Chinese pregnant women. BJOG.

[8] Qiu C, Enquobahrie D, Frederick I, Abetew D, Williams M (2010). Glucose intolerance and gestational diabetes risk in relation to sleep duration and snoring during pregnancy: A pilot study. BMC Womens Health..

[9] Bourjeily G, Raker CA, Chalhoub M, Miller MA (2010). Pregnancy and fetal outcomes of symptoms of sleep-disordered breathing. Eur. Respir. J..

[10] Facco FL, Grobman WA, Kramer J, Ho KH, Zee PC (2010). Self-reported short sleep duration and frequent snoring in pregnancy: Impact on glucose metabolism. Am. J. Obstet. Gynecol..

[11] Facco FL, Lappen J, Lim C, Zee PC, Grobman WA (2013). Preeclampsia and sleepdisordered breathing: A case-control study. Pregnancy Hypertens..

[12] Louis J (2012). Perinatal outcomes associated with obstructive sleep apnea in obese pregnant women. Obstet. Gynecol..

[13] Lyton DM (2013). Treatment of sleep disordered breathing reverses low fetal activity levels in preeclampsia. Sleep.

[14] O'Brien LM (2012). Pregnancy-onset habitual snoring, gestational hypertension, and preeclampsia: Prospective cohort study. Am. J. Obstet. Gynecol..

[15] Yinon D (2006). Pre-eclampsia is associated with sleep-disordered breathing and endothelial dysfunction. Eur. Respir. J..

[16] Louis JM, Mogos MF, Salemi JL, Redline S, Salihu HM (2014). Obstructive sleep apnea and severe maternal-infant morbidity/mortality in the United States, 1998–2009. Sleep.

[17] Warland J, Dorrian J, Morrison JL, O'Brien LM (2018). Maternal sleep during pregnancy and poor fetal outcomes: A scoping review of the literature with meta-analysis. Sleep Med. Rev..

[18] Yin TT (2008). Hypertension, fetal growth restriction and obstructive sleep apnoea in pregnancy. Eur. J. Obstet. Gynecol. Reprod. Biol..

[19] Ayrim A (2011). Influence of self-reported snoring and witnessed sleep apnea on gestational hypertension and fetal outcome in pregnancy. Arch. Gynecol. Obstet..

[20] Tauman R, Sivan Y, Katsav S, Greenfeld M, Many A (2011). Maternal snoring is not associated with fetal growth restriction. J. Matern. Fetal Neonatal Med..

[21] Franklin KA (2000). Snoring, pregnancy-induced hypertension, and growth retardation of the fetus. Chest.

[22] Bourjeily G, Chalhoub M, Miller MA (2010). Pregnancy and fetal outcomes of symptoms of sleep disordered breathing. Eur. Respir. J..

[23] Louis JM, Auckley D, Sokol RJ, Mercer BM (2010). Maternal and neonatal morbidities associated with obstructive sleep apnea complicating pregnancy. Am. J. Obstet. Gynecol..

[24] Chen YH (2012). Obstructive sleep apnea and the risk of adverse pregnancy outcomes. Am. J. Obstet. Gynecol..

[25] Telerant A, Dunietz GL, Many A, Tauman R (2018). Mild maternal obstructive sleep apnea in non-obese pregnant women and accelerated fetal growth. Sci. Rep..

[26] Song SO (2019). Metabolic consequences of obstructive sleep apnea especially pertaining to diabetes mellitus and insulin sensitivity. Diabetes Metab. J..

[27] Keenan K (2017). Concordance between maternal recall of birth complications and data from obstetrical records. Early Hum. Dev..

[28] O'Brien LM (2012). Validation of watch-PAT-200 against polysomnography during pregnancy. J. Clin. Sleep Med..

[29] Pittman SD (2004). Using a wrist worn device based on peripheral arterial tonometry to diagnosed obstructive sleep apnea: In laboratory and ambulatory validation. Sleep.

[30] American Academy of Sleep Medicine Task Force (1999). Sleep-related breathing disorders in adults: Recommendation for syndrome definition and measurement techniques in clinical research. Sleep.

[31] Matthews DR (1985). Homeostasis model assessment: Insulin resistance and beta-cell function from fasting plasma glucose and insulin concentrations in man. Diabetologia.

[32] Endo S (2006). Differences in insulin sensitivity in pregnant women with overweight and gestational diabetes mellitus. Gynecol. Endocrinol..

[33] Sierra-Laguado J (2007). Determination of insulin resistance using the homeostatic model assessment (HOMA) and its relation with the risk of developing pregnancy-induced hypertension. Am. J. Hypertens..

[34] Freedman DS (2017). Trends in weight-for-length among infants in WIC from 2000 to 2014. Pediatrics.

[35] Kuczmarski RJ (2002). 2000 CDC Growth Charts for the United States: Methods and development. Vital Health Stat..

[36] Addo OY, Himes JH (2010). Reference curves for triceps and subcapsular skinfold thickness in US children and adolescents. Am. J. Clin. Nutr..

[37] Crume EL (2011). Long term impact of neonatal breastfeeding on childhood adiposity and fat distribution among children exposed to diabetes in utero. Diabetes Care.

[38] American College of Obstetricians and Gynecologists (2013). ACOG Committee opinion no. 548: Weight gain during pregnancy. Obstet. Gynecol..

[39] Kassi E, Pervanidou P, Kaltsas G, Chrousos G (2011). Metabolic syndrome: Definitions and controversies. BMC Med..

[40] Ryan S, Taylor CT, McNicholas WT (2005). Selective activation of inflammatory pathways by intermittent hypoxia in obstructive sleep apnea syndrome. Circulation.

[41] Okun ML, Coussons-Read M, Hall M (2009). Disturbed sleep is associated with increased CRP in young women. Brain Behav. Immun..

[42] Tauman R (2011). Maternal snoring during pregnancy is associated with enhanced fetal erythropoiesis—A preliminary study. Sleep Med..

[43] Dupont C (2018). The predictive value of head circumference growth during the first year of life on early child traits. Sci. Rep..

[44] Brunton PJ, Russell JA (2011). Neuroendocrine control of maternal stress responses and fetal programming by stress in pregnancy. Prog. Neuropsychopharmacol. Biol. Psychiatry..

[45] Miranda A, Sousa N (2018). Maternal hormonal milieu influence on fetal brain development. Brain Behav..

[46] Zerbo O (2016). Maternal mid-pregnancy C-reactive protein and risk of autism spectrum disorders: The early markers for autism study. Transl. Psychiatry..

[47] Tauman R (2015). The effect of maternal sleep-disordered breathing on the infant's neurodevelopment. Am. J. Obstet. Gynecol..

[48] Gluckman PD, Hanson MA, Cooper C, Thornburg KL (2008). Effect of in utero and early-life conditions on adult health and disease. N. Engl. J. Med..

[49] Khalyfa A (2017). Late gestational intermittent hypoxia induces metabolic and epigenetic changes in male adult offspring mice. J. Physiol..

[50] Ducsay CA (2018). Gestational hypoxia and developmental plasticity. Physiol. Rev..

[51] Horikoshi M (2016). Genome-wide associations for birth weight and correlations with adult disease. Nature.

[52] Robertson NT, Turner JM, Kumar S (2019). Pathophysiological changes associated with sleep disordered breathing and supine sleep position in pregnancy. Sleep Med. Rev..

[53] King K, Murphy S, Hoyo C (2015). Epigenetic regulation of Newborns' imprinted genes related to gestational growth: Patterning by parental race/ethnicity and maternal socioeconomic status. J. Epidemiol. Community Health..

