# Author Correction: Mild maternal sleep-disordered breathing during pregnancy and offspring growth and adiposity in the first 3 years of life

**DOI:** 10.1038/s41598-020-75084-8

**Published:** 2020-11-09

**Authors:** Avivit Brener, Yael Lebenthal, Sigal Levy, Galit Levi Dunietz, Orna Sever, Riva Tauman

**Affiliations:** 1 Pediatric Endocrinology and Diabetes Unit, Dana-Dwek Children’s Hospital, Tel Aviv Sourasky Medical Center, 6423906 Tel Aviv, Israel; 2 Sackler Faculty of Medicine, Tel Aviv University, 6997801 Tel Aviv, Israel; 3 Statistical Education Unit, The Academic College of Tel Aviv Yaffo, 6818211 Tel Aviv, Israel; 4 Sleep Disorders Center, Department of Neurology, University of Michigan, Ann Arbor, MI USA; 5 Sleep Disorders Center, Tel Aviv Sourasky Medical Center, 6 Weizman Street, 6423906 Tel Aviv, Israel

Correction to: *Scientific Reports* 10.1038/s41598-020-70911-4, published online 19 August 2020

This original version of this Article contained errors.

In the original version, the author Galit Levi Dunietz was incorrectly indexed.

In Figure 1, the description for “Laboratory evaluation” was incomplete.

“Blood samples for fasting glucose, insulin, HbA1c, and CRP were drawn on the morning following the overnight sleep study”

now reads:

"Blood samples for fasting glucose, insulin, HbA1c, lipid panel and CRP were drawn on the morning following the overnight sleep study"

In Figure 2C, the colour of the lines used to identify “offspring of mothers with mild SDB” and “offspring of mothers without SDB” was erroneously inverted.

These errors have now been corrected in the PDF and HTML versions of the Article.

